# Poplar-based thermochromic composites that change colour at 38 °C to 46 °C

**DOI:** 10.1038/s41598-021-95274-2

**Published:** 2021-08-19

**Authors:** Weihua Zou, Zimu Li, Zhangheng Wang, Delin Sun, Pingfang Zhang

**Affiliations:** Central South University of Forestry and Technology, Shaoshan South Road 498, Changsha, 410004 China

**Keywords:** Materials science, Materials for devices, Structural materials

## Abstract

The red thermochromic dye (R-TD) is the tetradecanoic acid tetradecyl ester (C_28_H_56_O_2_) and methyl red (C_15_H_15_N_3_O_2_) mixture that has better permeability enabling its infiltration into wood and better thermochromic properties changing its colour at above 30 °C after about 0.5 min. Thicker poplar-based thermochromic composite specimens (R-PTC, thickness: 5.0 mm) were prepared by filling the R-TD into pre-treated poplar veneer (thickness: 5.0 mm) thus allowing better penetration after pre-treatment. After R-TD infiltration, the R-PTC samples were covered by polypropylene wax for preventing R-TD from overflowing from R-PTC under the action of phase-change temperature. This R-PTC, whose colour can change from light-red to dark-red at 38 °C to 46 °C, can recover to light-red at below 38 °C after about 14 h, and the peak of colour change is at about 42 °C. R-PTC will be suitable for materials used in thermochromic furniture that can indicate the surface temperature to potential users, thus allowing assessment of likely scalded pain when used the furniture.

## Introduction

Thermochromic wood, as a thermochromic material, can undergo a reversible change in colour with respect to temperature stimulus^1–5^, that has the advantages of renewability, low cost, easy preparation, easy modification and so on. Thermochromic wood can be used in many fields, such as: temperature sensors^6–8^, smart window frame^9–11^, temperature control coatings^12,13^, thermal energy storage^14^, camouflage^15^, etc. Infiltrating thermochromic dyes into wood materials will help to improve the seasonal visual effect of wood, that can enrich the decorative effect of wood materials, can be used as temperature indicators, and can provide a new solution to energy consumption for interior buildings^16,17^. Professor Fu Feng and coworkers have investigated the development of thermochromic wood^18^, the preparation and properties of thermochromic thin wood-based veneer (thickness: 0.7 mm)^19–21^, and multi-functional thermochromic energy-storage wood materials^22^. Xiaodong Zhu and coworkers have prepared a thin thermochromic wood-based veneer (thickness: 0.17 mm) that can be obtained after impregnation with a thermochromic compound suspension for 2.0 min at 65 °C^16^.

Poplar is mainly distributed from low altitudes to 4800 m above sea level, and between latitudes 22° to 70° N^23–25^. Farmed poplar is an important agro-forestry tree species in many nations due to its rapid growth, short rotation on stand, multiple uses, and high economic value^26–29^. Reasonable use of farmed poplar can meet the human demand for thermochromic materials and avoid the consumption of natural forest resources.

**Figure 1:**
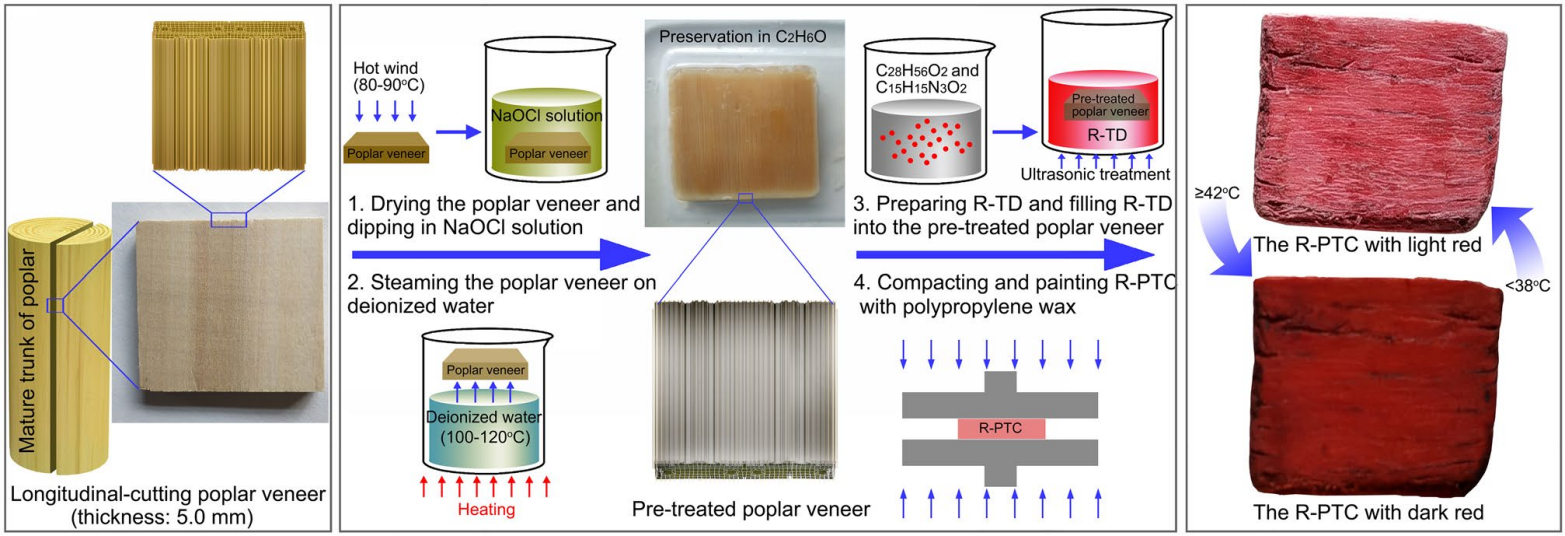
Preparation of R-PTC.

In the present research, a thicker, poplar-based, thermochromic composite (R-PTC, thickness: 5.0 mm) was prepared by infiltration of red thermochromic dyes (R-TD) into the pre-treated poplar veneer (thickness: 5.0 mm) and compacting the resulting poplar-based composite (Fig. 1). This type of R-PTC can undergo a colour change from light-red to dark-red at about 38 °C to 46 °C, can revert to light-red at below 38 °C after about 14 h, and the peak of colour change is at about 42 °C.

## Results and discussion

**Figure 2:**
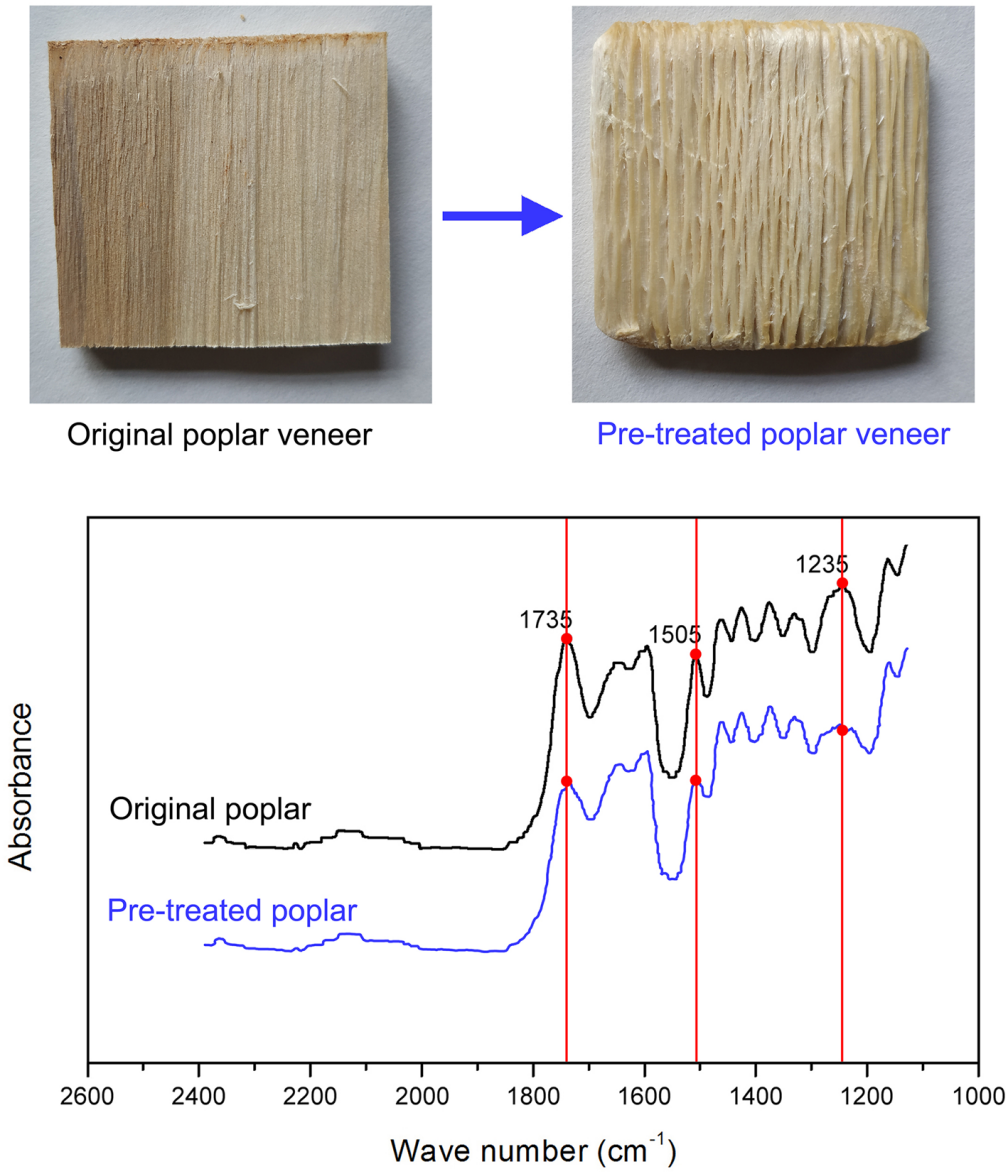
FTIR spectra: original, and pre-treated poplar specimens.

Fourier transform infrared spectroscopy (FTIR) was used to investigate changes in the composition of the cell wall of poplar specimens before and after pre-treatment. FTIR spectra were measured using an FTIR-850 (Gangdong, Tianjin, China). In the FTIR spectrum, the band at 1505 cm^−1^ represents the aromatic skeleton vibration of the lignin^28–31^. The band at 1235 cm^−1^ represents the characteristic vibration of hemicelluloses, and that at 1735 cm^−1^ signifies the presence of a C=O functional group^32–35^. The 1505 cm^−1^, 1235 cm^−1^, and 1735 cm^−1^ peaks represent lignin, hemicelluloses, and C=O functional group, respectively. After pre-treatment, the 1505 cm^−1^, 1235 cm^−1^, and 1735 cm^−1^ peaks of pre-treated poplar specimens are lower than the peaks of the original poplar specimens in the FTIR spectra (Fig. 2), proving that lignin, hemicellulose, and the C=O functional group may had been changed therefrom. As Table 1 shows, the absolute-dry mass of original poplar specimens (60 mm × 60 mm × 5 mm) was about 4.244 ~ 4.391 g, and the absolute-dry mass of pre-treated poplar specimens (60 mm × 60 mm × 5 mm) was about 3.113–3.382 g, and the absolute-dry mass of pre-treated poplar specimens was less than that of the original poplar specimens, with the mass being reduced by about one-third of its original value after pre-treatment. Therefore, to compare with the original poplar specimens, the lignin, hemicellulose, and the C=O functional group may had been largely removed in the pre-treated poplar specimens.

**Table 1:** The absolute-drying masses: original, and pre-treated poplar specimens.

	Sample 1 (g)	Sample 2 (g)	Sample 3 (g)
Absolute-drying mass: original poplar (60 mm × 60 mm × 5 mm)	4.244	4.362	4.391
Absolute-drying mass: pre-treated poplar (60 mm × 60 mm × 5 mm)	3.113	3.214	3.382

**Figure 3:**
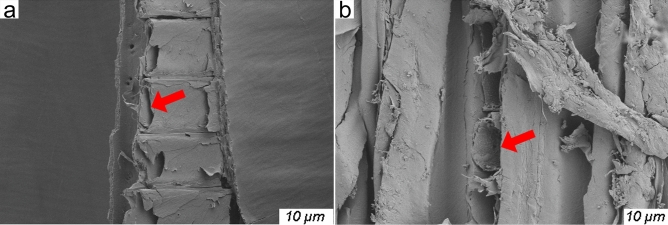
SEM images: original, and pre-treated poplar specimens.

Original, and pre-treated poplar specimens were cut from the longitudinal direction, these sections were examined by using Sigma 300 scanning electron microscopy (Zeiss, Germany). Figure 3a,b are SEM images of longitudinal direction from original, and pre-treated poplar specimens, respectively. As the red arrows show (Fig. 3), the cell cavity space was obviously enlarged after pre-treatment, that improved the channel of R-TD infiltration.

**Figure 4:**
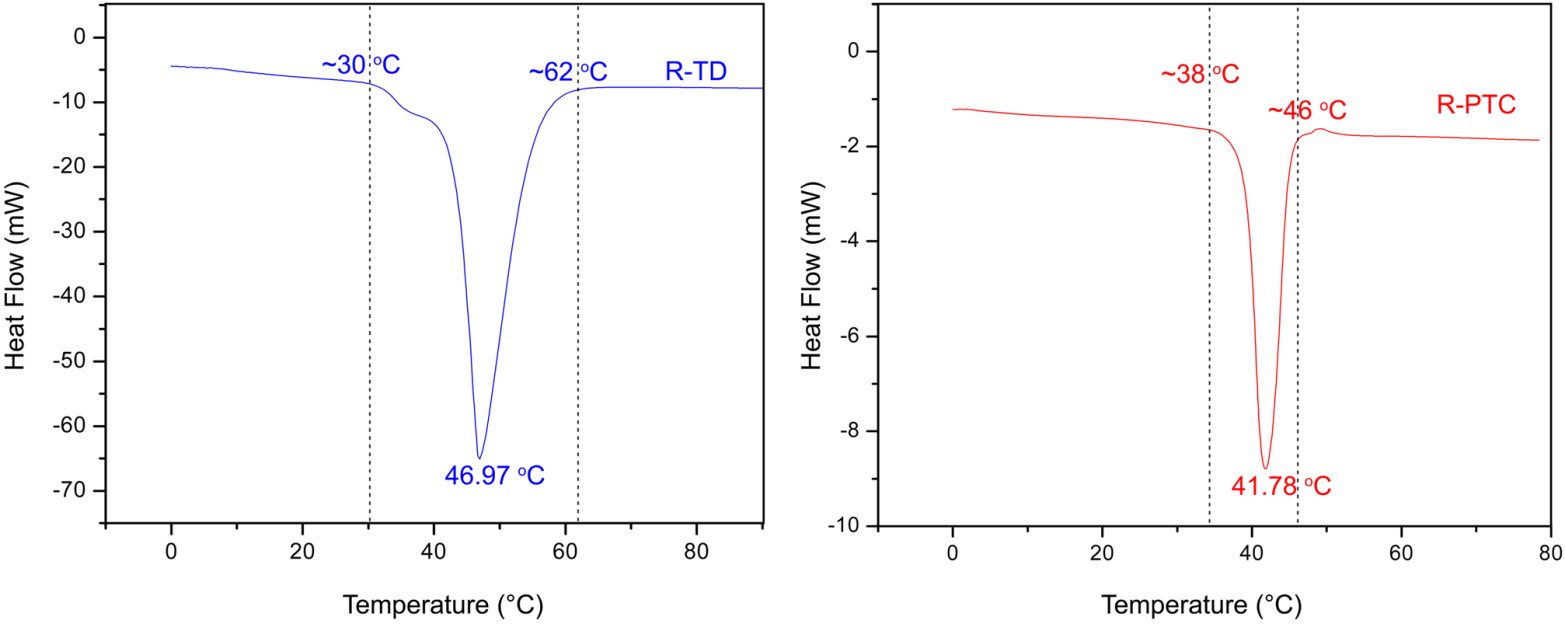
The DSC curve of R-TD and R-PTC.

DSC test of R-TD and R-PTC. The phase-change temperature of thermochromic dyes can affect the temperature during dye-infiltration and the colour-change temperature of a thermochromic material^19,20^. A differential scanning calorimeter (DSC) can be used to measure phase-change temperature (Fig. 4): the peak value of phase-change temperature was 46.97 °C in R-TD, and the peak value was 41.78 °C in R-PTC. As Fig. 4 shows, the phase-change temperature range of R-TD is about 30 °C to 62 °C, and the phase-change temperature range of R-PTC is about 38 °C to 46 °C. In R-PTC, the covalent bond between wood fibers and R-TD that potentially improved the starting temperature of phase-change temperature range, and the water molecule of wood that possibly reduced the peak value and range of phase-change temperature. Therefore, comparing with R-TD, the R-PTC had lower peak value of phase-change temperature, and narrower phase-change temperature range.

**Figure 5:**
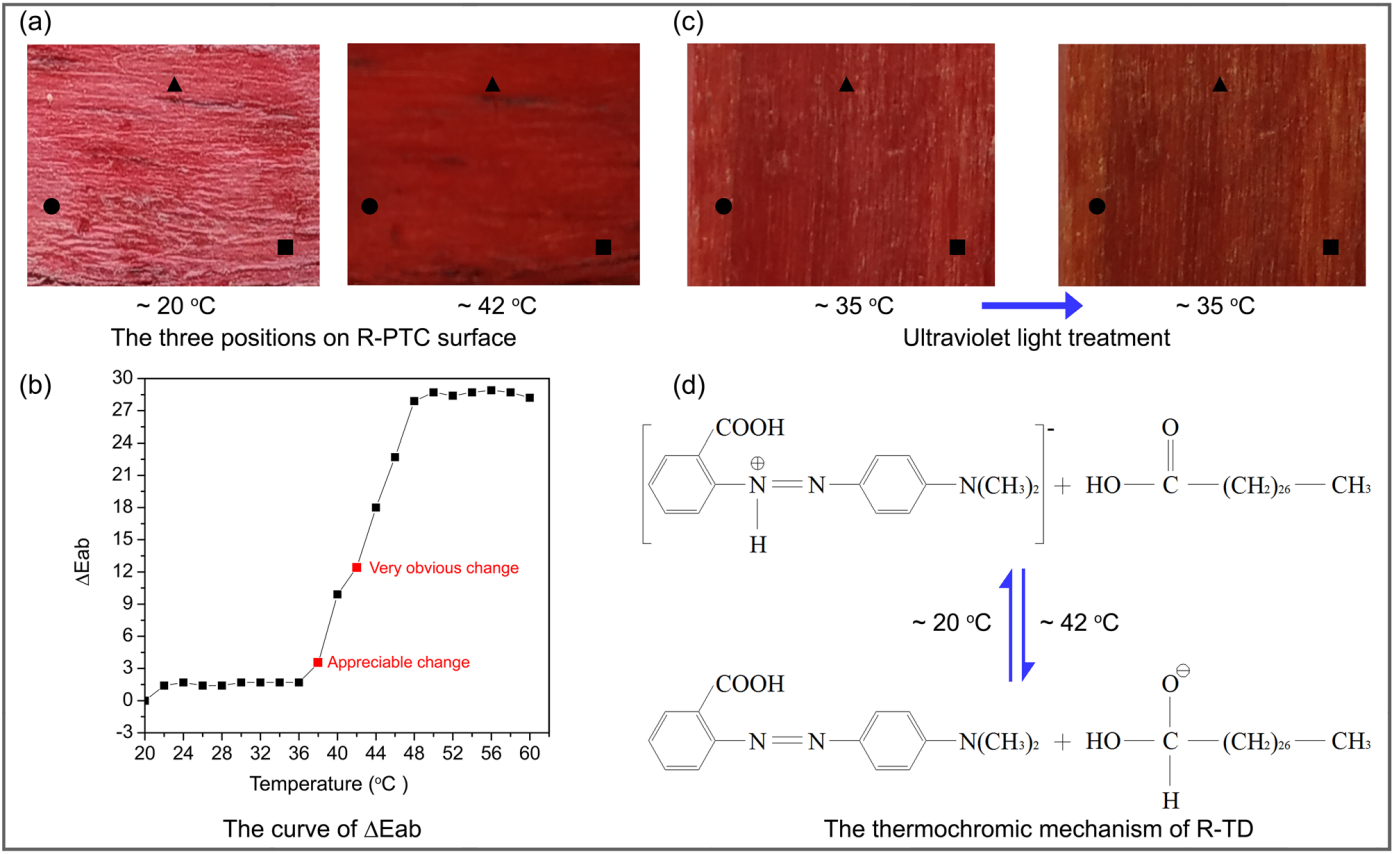
(**a**) The colours of the R-PTC surface changed from light-red (below 38 °C) to dark-red (above 42 °C). (**b**) The curve of *∆E*_*ab*_. (**c**) The colours of the R-PTC surface changed before and after ultraviolet light treatment. (**d**) The thermochromic mechanism of R-TD.

Thermochromic properties of R-PTC. According to CIELab measurements (International Lighting Committee, 1976) and the environmental temperature range of R-PTC, the R-PTC specimens were placed into a constant-temperature box for about 10 min at a specific temperature from 20 to 60 °C (Table 2), and the colour parameters (*L*, *a*, and *b*) of R-PTC surface were measured using a CR-400 colorimeter (Konica Minolta, Japan) at each specific temperature (Table 2). Each colour parameter is the average value of three measurements at different positions (●, ■, and ▲) on the R-PTC surface (Fig. 5a). In Table 2, *∆E*_*ab*_ is the value of the colour difference, and the magnitude of *∆E*_*ab*_ affects the strength of human vision (Table 3)^36,37^; *∆E*_*ab*_ is given by:

$$ \Delta E_{ab} = \sqrt {(L_{n} - L_{0} )^{2} + (a_{n} - a_{0} )^{2} + (b_{n} - b_{0} )^{2} } $$

*L*_0_, *a*_0_, and *b*_0_ are the colour parameters of the R-PTC surface at 20 °C; *L*_*n*_, *a*_*n*_, and *b*_*n*_ are the colour parameters of the R-PTC surface at 22 °C, 24 °C, 26 °C, …, 58 °C, and 60 °C, respectively.

**Table 2:** The colour parameters of the R-PTC surface at temperatures from 20 to 60 °C.

Temperature (°C)	L:	a:	b:	*∆E*_*ab*_
20	52	43	18	0
22	51	42	18	1.4
24	53	42	17	1.7
26	53	44	18	1.4
28	51	43	19	1.4
30	51	44	19	1.7
32	51	44	17	1.7
34	53	42	17	1.7
36	53	44	19	1.7
38	54	45	20	3.5
40	47	48	25	9.9
42	44	48	26	12.4
44	40	49	30	18
46	38	51	34	22.7
48	35	50	39	27.9
50	27	43	32	28.7
52	28	41	33	28.4
54	26	44	30	28.7
56	28	42	34	28.9
58	27	42	32	28.7
60	27	42	31	28.2

**Table 3:** The relation between *∆E*_*ab*_ and identification ability of human vision.

*∆E*_*ab*_	Human vision
0–0.5	Unnoticeable change
0.5–1.5	Slight change
1.5–3.0	Appreciable change
3.0–6.0	Recognition change
6.0–12.0	Obvious change
> 12.0	Very obvious change

As shown in Table 2 and Fig. 5b, at 38 °C to 42 °C, *∆E*_*ab*_ exceeded 3.0 and gradually reached 12 (Table 2). Data in Table 3 show that the colour differences in R-PTC changed at 38 °C and did so to a much greater extent at 42 °C to the human eye. In Fig. 5a, the colour of the R-PTC surface is seen to have changed from light-red (at < 38 °C) to dark-red (at ≥ 42 °C). The thermochromic properties of R-PTC basically accords with the DSC test of R-PTC, the peak value all appeared at about 42 °C, and the range all appeared at about 38 °C to 46 °C.

After heating the R-PTC specimens in a constant-temperature box (Symor, Heifei, China) for about 150 h at 80 to 100 °C, the weight of heat-treated R-PTC was reduced about 21% of original R-PTC. As Fig. 5c shown, at ●, ■, and ▲ positions on the R-PTC surface, the colour parameter of three positions were measured before and after the R-PTC specimens were exposed to ultraviolet light (120 mw/cm^2^) for about 150 h, and its *∆E*_*ab*_ was 5.7, recognition change.

The colour of methyl red (C_15_H_15_N_3_O_2_) is red and acid form (HMR) when its pH value was ≤ 4.4 and its wavelength was at 520 nm, and the colour of methyl red (C_15_H_14_N_3_O_2_) is yellow and alkaline form (MR) when its pH value was ≥ 6.2 and its wavelength was at 430 nm^38–41^. R-TD is the tetradecanoic acid tetradecyl ester (C_28_H_56_O_2_) and methyl red (C_15_H_15_N_3_O_2_) mixture. In R-TD, C_28_H_56_O_2_ is the hide-colour solvent, and C_15_H_15_N_3_O_2_ is the developer. As Fig. 5a,d shown, the R-TD was light-red and its developer was acid form (HMR) at about 20 °C, and the R-TD was dark-red and its developer was alkaline form (MR) at about 42 °C.

**Figure 6:**
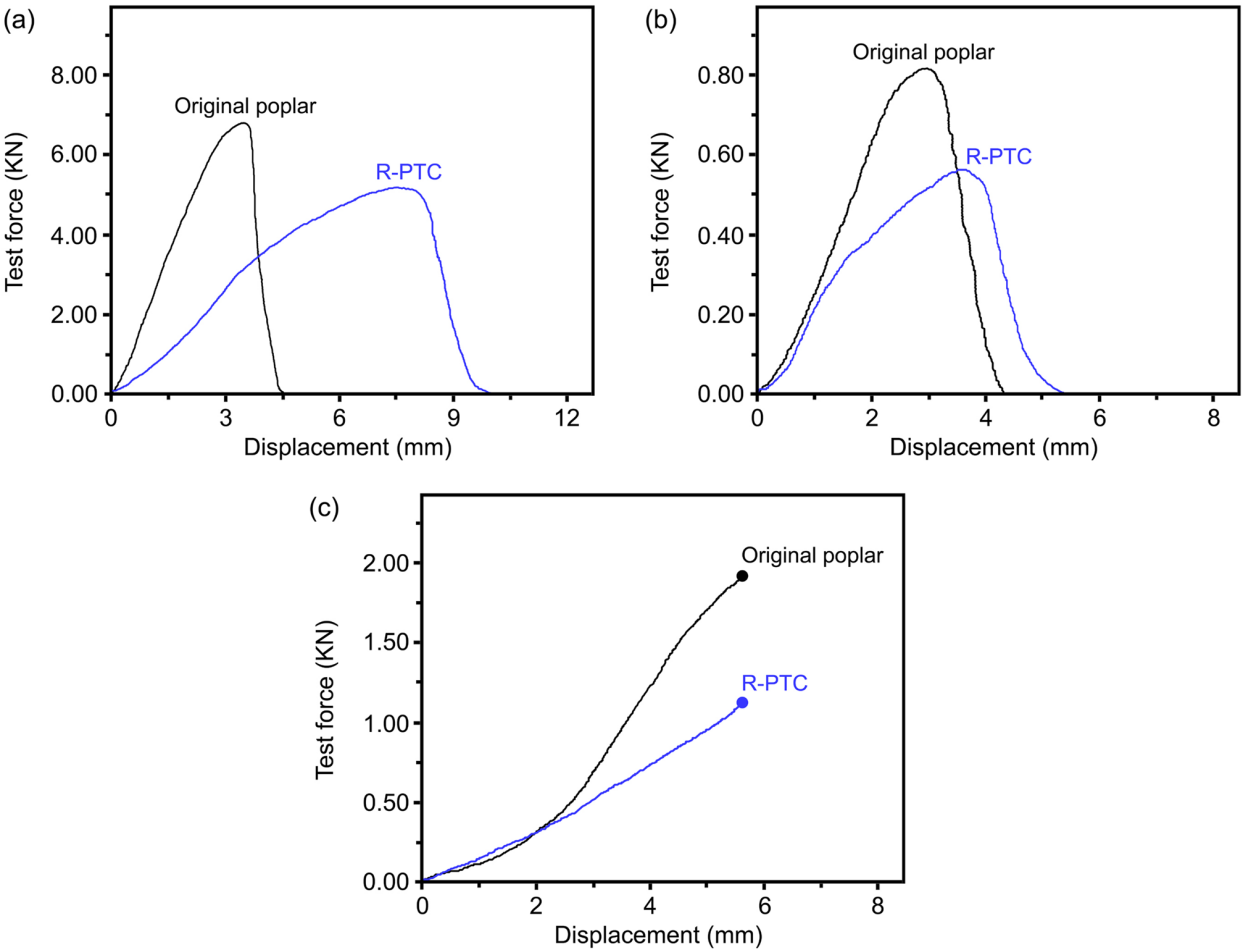
(**a**) Longitudinal compressive strength. (**b**) Longitudinal tensile strength. (**c**) Radial hardness.

Mechanical characteristics of R-PTC. The longitudinal compressive strength and tensile strength, and radial hardness of R-PTC were measured. Compared to the original poplar specimens, the R-PTC had a slightly lower longitudinal compressive strength, tensile strength, and radial hardness (Fig. 6). The mechanical characteristics of pre-treated poplar were reduced after the loss of the lignin during pre-treatment: this also influenced the mechanical characteristics of the R-PTC specimens, therefore, improving the mechanical characteristics of R-PTC without affecting its thermochromic properties will form a focus of future research.

**Figure 7:**
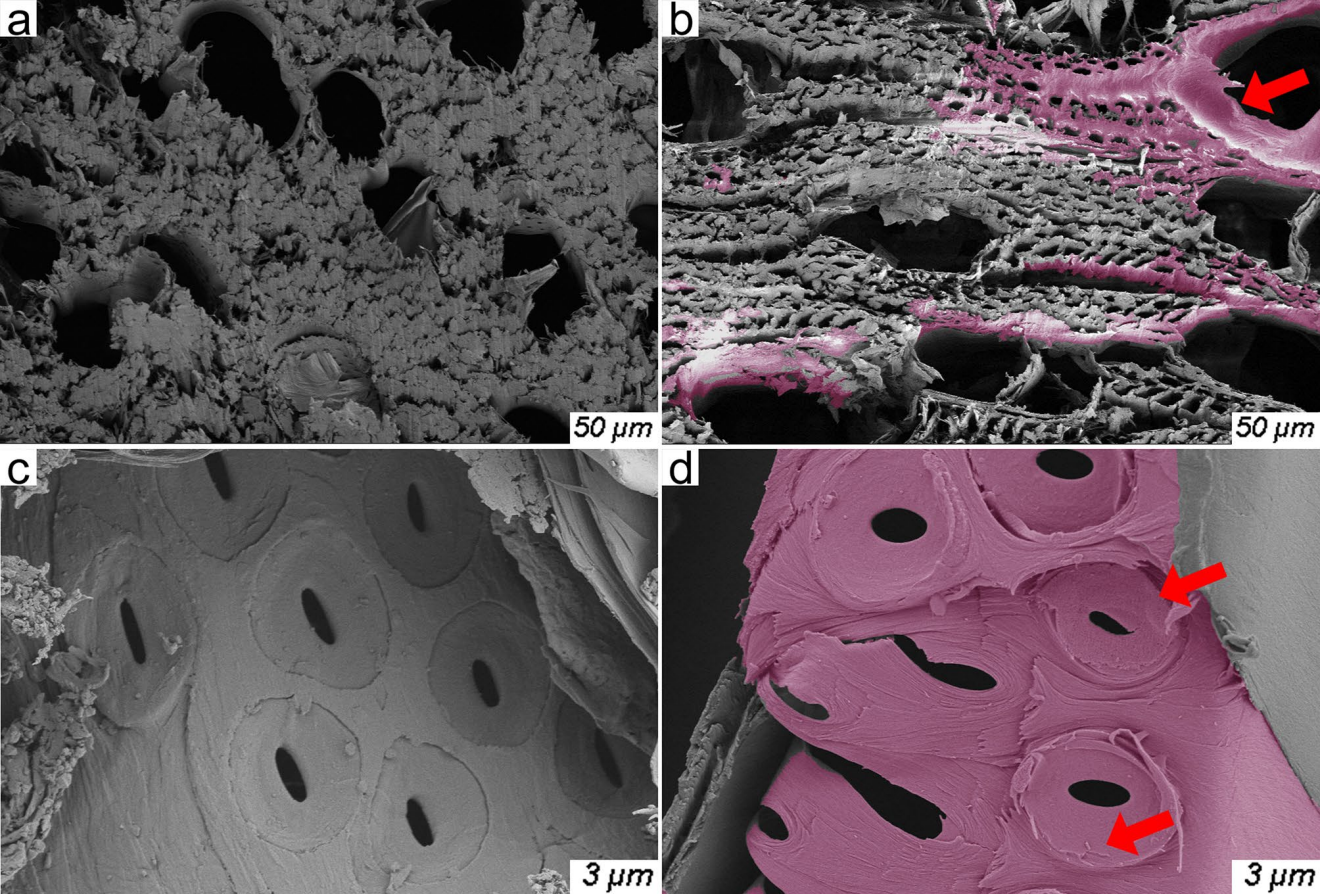
SEM images: pre-treated poplar specimens (**a**, **c**) and R-PTC specimens (**b**, **d**).

**Figure 8:**
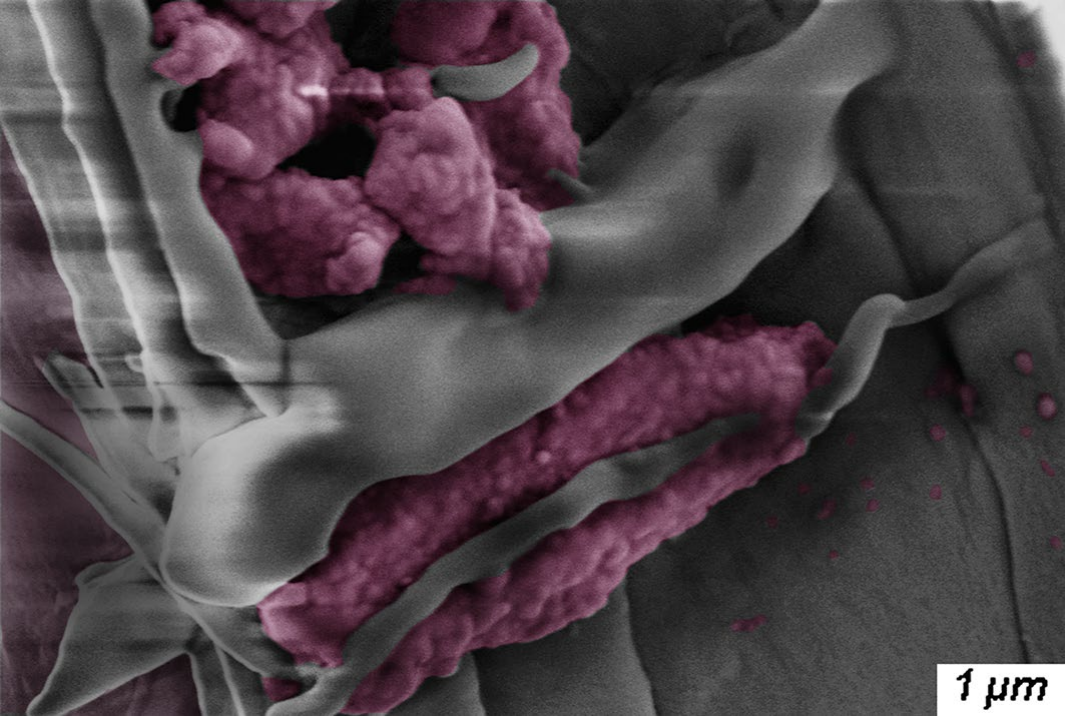
SEM image: the R-TD in R-PTC specimen.

Microscopic features of R-PTC. The microscopic features of R-PTC were examined by SEM. Figure 7a,c show SEM images of radial-cut and longitudinal-cut pre-treated poplar specimens, respectively. Figure 7b,d show SEM images of radial-cut and longitudinal-cut R-PTC, respectively. Compared with Fig. 7a,c,b,d show that the R-PTC was able to be infiltrated by R-TD, which as the red arrows show, R-TD had been attached to the cell wall of wood. In R-PTC, the R-TD gathered around the fibers and on the surface of cell wall, and the R-TD liked irregular ball-like object that combined with the fibers of wood (Fig. 8).

## Conclusions

The colour of the newly-developed R-PTC could change from light-red to dark-red at 38 °C to 46 °C, can gradually revert to light-red at below 38 °C after about 14 h, and the peak of colour change is at about 42 °C. When the temperature of object at ≥ 42 °C, the user will feel scalded pain after touching the object. Therefore, R-PTC could be used in material used to make furniture capable of indicating the surface temperature to potential users, allowing them to assess likely scalded pain. Furthermore, future work will focus on improvement of its mechanical characteristics.

### Experimental

Materials and chemicals. The original poplar (60 mm × 60 mm × 5 mm) was purchased from Mudan Wood Co., Ltd. (Suqian, China). Sodium hypochlorite (NaOCl, > 98%), deionised water, and absolute ethyl alcohol (C_2_H_6_O, > 99.5%) were purchased from Aladdin Biochemical Technology (Shanghai, China). Tetradecanoic acid tetradecyl ester (C_28_H_56_O_2_, > 99%) and polypropylene wax were purchased from Shandong Usolf Chemical Technology Co., Ltd. (Linyi, China). Methyl red (C_15_H_15_N_3_O_2_, > 99%) was purchased from Fangzheng Reagent Factory (Tianjin, China).

The preparation process of R-PTC included pre-treatment of the specimens, preparation of R-TD, R-TD infiltration, and compaction (Table 4).

**Table 4:** The chemical formula and method of preparation of R-PTC.

Treatment method (s)	Chemicals (g, ml)	Temperature (°C)	Time (h)
Pre-treatment of Sample 1 (dipping sample)	NaOCl (30 g), Deionised water (500 ml)	15–25	48
Pre-treatment of sample 2 (steaming sample)	Deionised water (2500 ml)	100–120	6
Preparation of R-TD and R-TD infiltration	C_28_H_56_O_2_ (10 g), C_15_H_15_N_3_O_2_ (0.1 g)	75–80	2.5
Compaction and painting treatment		15–25	0.5

Sample pre-treatment. After drying the original poplar specimens in a constant-temperature box (Symor, Heifei, China) for about 3 h at 80 to 90 °C, the sample was dipped in NaOCl solution (0.81 mol L-1 in deionised water) for about 48 h at 15 to 25 °C. Then, the specimens were steamed over deionised water for about 6 h at 100 to 120 °C. After repeating these pre-treatment, all added chemicals were removed from the specimens by rinsing in hot distilled water, and subsequent preservation in C_2_H_6_O.

Preparation of R-TD and R-TD infiltration. R-TD was prepared by mixing C_28_H_56_O_2_ (10 g) and C_15_H_15_N_3_O_2_ (0.1 g) for about 0.5 h at about 75 to 80 °C. The pre-treated poplar specimens were then removed from the C_2_H_6_O and dried in a constant-temperature box for about 1 h at 80 to 90 °C. The resulting R-TD was infiltrated into the lumen of pre-treated poplar sample by R-TD impregnation treatment in a beaker under ultrasonication in a DR-LQ20D ultrasonic cleaner (Derui, Shenzheng, China) for about 2 h at 75 to 80 °C, and an applied ultrasonic power of 80 W.

Compaction and painting treatment. After R-TD infiltrating the sample, the poplar-based thermochromic composite (R-PTC) specimens were compacted in a 150 T universal test press (Suzhou, Shanghai, China) under an applied stress of 4.5 MPa for about 0.5 h at 15 to 25 °C, and the R-PTC samples were covered by polypropylene wax for preventing R-TD from overflowing from R-PTC under the action of phase-changed temperature.

Statement. All methods were performed in accordance with the relevant guidelines and regulations.
